# Supplementary material for: Probiotic supplementation reduces inflammatory profiles but does not prevent oral immune perturbations during SIV infection
Source: Sci Rep. 2021 Jul 15;11:14507. doi: 10.1038/s41598-021-93918-x (PMC8282626; doi:10.1038/s41598-021-93918-x)
Supplement: Supplementary file 1 — Supplementary Information. [file 41598_2021_93918_MOESM1_ESM.docx]

**Probiotic Supplementation Reduces Inflammatory Profiles But Does Not Prevent Oral Immune Perturbations During SIV Infection**

Rhianna Jones^1^, Kyle Kroll^1^, Courtney Broedlow^2,3^, Luca Schifanella^2^, Scott Smith^1^, Brady Hueber^1^, Spandan V. Shah^1^, Daniel R. Ram^1^, Cordelia Manickam^1^, Valerie Varner^1^, Nichole R. Klatt^2,3^, R. Keith Reeves^1,4*^

- ^1^Center for Virology and Vaccine Research, Beth Israel Deaconess Medical Center, Harvard Medical School, Boston, MA; ^2^ Division of Surgical Outcomes and Precision Medicine Research, Department of Surgery, University of Minnesota, Minneapolis, MN, USA; ^3^Department of Pharmaceutics, University of Washington, Seattle, WA, USA; ^4^Ragon Institute of Massachusetts General Hospital, MIT, and Harvard, Cambridge, MA 02139, USA

**Supplementary Information**

**Supplementary Figure 1**

**Supplementary Figure 1. Representative flow cytometry gating.**

Representative gating strategies are shown for (A) NKG2A/C+ NK cells and NKp44+ ILC3, (B) T cells, and (C) T cell phenotypes.

**Supplementary Figure 2. PCA of Luminex analyte data.**

| **Target** | **Fluorochrome** | **Clone** | **Vendor** |
| --- | --- | --- | --- |
| Caspase-3 | Alexa647 | C92-605 | BD Pharmingen |
| CD3 | APC CY7 | SP34.2 | BD Pharmingen |
| CD4 | FITC | L200 | BD Pharmingen |
| CD8 | PAC BLUE | RPA-T8 | BD Pharmingen |
| CD8 | PE CY7 | RPA-T8 | BD Pharmingen |
| CD11C | A700 | 3.9 | Affymetrix |
| CD14 | BV650 | M5E2 | BD Pharmingen |
| CD16 | BUV496 | 3G8 | BD Pharmingen |
| CD20 | BUV395 | L27 | BD Pharmingen |
| CD28 | PE CF594 | 28.2 | BD Pharmingen |
| CD45 | BV605 | D058-1283 | BD Pharmingen |
| CD56 | BV786 | NCAM16.2 | BD Pharmingen |
| CD62L | BV711 | SK11 | BD Pharmingen |
| CD95 | BV421 | DX2 | BD Pharmingen |
| CD123 | PE CY7 | 7G3 | BD Pharmingen |
| CD159A | FITC | REA110 | MILTENYI |
| CD195 | PE | 3A9 | BD Pharmingen |
| CD197 | BUV395 | 150503 | BD Pharmingen |
| CD279 | BV786 | EH12.1 | BD Pharmingen |
| CD336 | PE | Z231 | BECKMAN COULTER |
| HLA-DR | ECD | IMMU-357 | BECKMAN COULTER |
| Ki-67 | PERCP CY5.5 | B56 | BD Pharmingen |

**Supplementary Table 1.** Antibodies used in this study

|  | **Day 28** | **Day 42** | **Day 113** |
| --- | --- | --- | --- |
| IL6 | 0.818 | 0.699 | 0.310 |
| CXCL13 | 0.004 | 0.261 | 1.00 |
| MIP1b | 0.132 | 0.093 | 0.485 |
| IP10 | 0.041 | 0.485 | 0.132 |
| IL1Ra | 0.132 | 0.240 | 0.589 |
| Perforin | 0.309 | 0.394 | 0.485 |
| IFN-alpha | 0.125 | 0.537 | 0.792 |
| IFN-gamma | 0.484 | 0.818 | 0.589 |
| MCP1 | 1.00 | 0.571 | 0.786 |
| MIG | 0.109 | 0.589 | 0.026 |
| IL-23 | 0.537 | 0.699 | 0.537 |
| MIF | 0.699 | 0.485 | 0.179 |

**Supplementary Table 2.** Mann-Whitney *U* test *p*-values comparing log2 fold change values between Control and Probiotic groups for each cytokine measured by Luminex assay.
